# Seroepidemiologic Study of Oropouche Virus, Amazonas State, Brazil, 2015–2016

**DOI:** 10.3201/eid3204.250917

**Published:** 2026-04

**Authors:** Julia Forato, Gabriel C. Scachetti, Bárbara B. Salgado, Carolina M.L. Singh, Nadielle C. Pereira, Renato dos Santos Reis, William M. de Souza, José Luiz Proenca-Modena, Pritesh Lalwani

**Affiliations:** University of Campinas, Campinas, Brazil (J. Forato, G.C. Scachetti, J.L. Proenca-Modena); Fiocruz Amazônia, Manaus, Brazil (B.B. Salgado, C.M.L. Singh, N.C. Pereira, P. Lalwani); Universidade Federal do Amazonas, Manaus (B.B. Salgado, C.M.L. Singh, N.C. Pereira, R. dos S. Reis, P. Lalwani); University of Kentucky, Lexington, Kentucky, USA (W.M. de Souza)

**Keywords:** Oropouche virus, viruses, Orthobunyavirus, Peribunyaviridae, serosurvey, vector-borne infections, zoonoses, arboviruses, Brazil

## Abstract

We conducted a cross-sectional serosurvey for Oropouche virus (OROV) among residents of Amazonas State, Brazil, during 2015–2016. We detected OROV neutralizing antibodies in 85/814 (10.4%) participants; seroprevalence was higher in Manaus (49/440 [11.1%]) than in Coari (36/374 [9.6%]). Those findings suggest OROV circulation in Amazonas State before 2015.

Oropouche virus (OROV) is a neglected and reemerging vectorborne orthobunyavirus endemic in Central and South America since the 1950s (*1*,*2*). OROV is mainly transmitted to humans by the bite of infected midges (*Culicoides paraensis*), which also spread OROV among sloths and other animals in the sylvatic cycle (*3*). OROV infection in humans causes Oropouche fever, characterized by self-limiting febrile illnesses but that can, in rare cases, lead to hemorrhagic, neurologic, and maternal–fetal complications, even death (*1*,*4*). 

OROV has been endemic in the Amazon region and expanding in 2024 toward areas of high population density in all Brazil states, as well as to the Caribbean, including Cuba, Barbados, and Panama (*5*). In addition, traveler-imported cases were identified in the United States, Canada, and Europe. Reports of OROV infection have been identified predominantly by molecular assays; OROV serosurvey studies remain scarce, even in OROV-endemic areas. In this study, we conducted a retrospective cross-sectional serosurvey among residents from Coari and Manaus municipalities in Amazonas State, Brazil, during 2015–2016. The ethics committee of the Federal University of Amazonas approved all procedures for this study (approval nos. 5.876.612 and 6.629.451).

We collected blood samples by venipuncture from residents in Manaus and Coari municipalities (Figure 1). No participants reported any symptoms in the 30 days before blood sample collection. We tested serum samples by 90% plaque reduction neutralization test (PRNT_90_) to assess the presence and titer of neutralizing antibodies against OROV (*5*). We determined PRNT_90_ values from the mean of 2 technical replicates by fitting a 3-parameter log-logistic regression curve to plaque counts normalized to the positive control (Appendix). 

**Figure 1:**
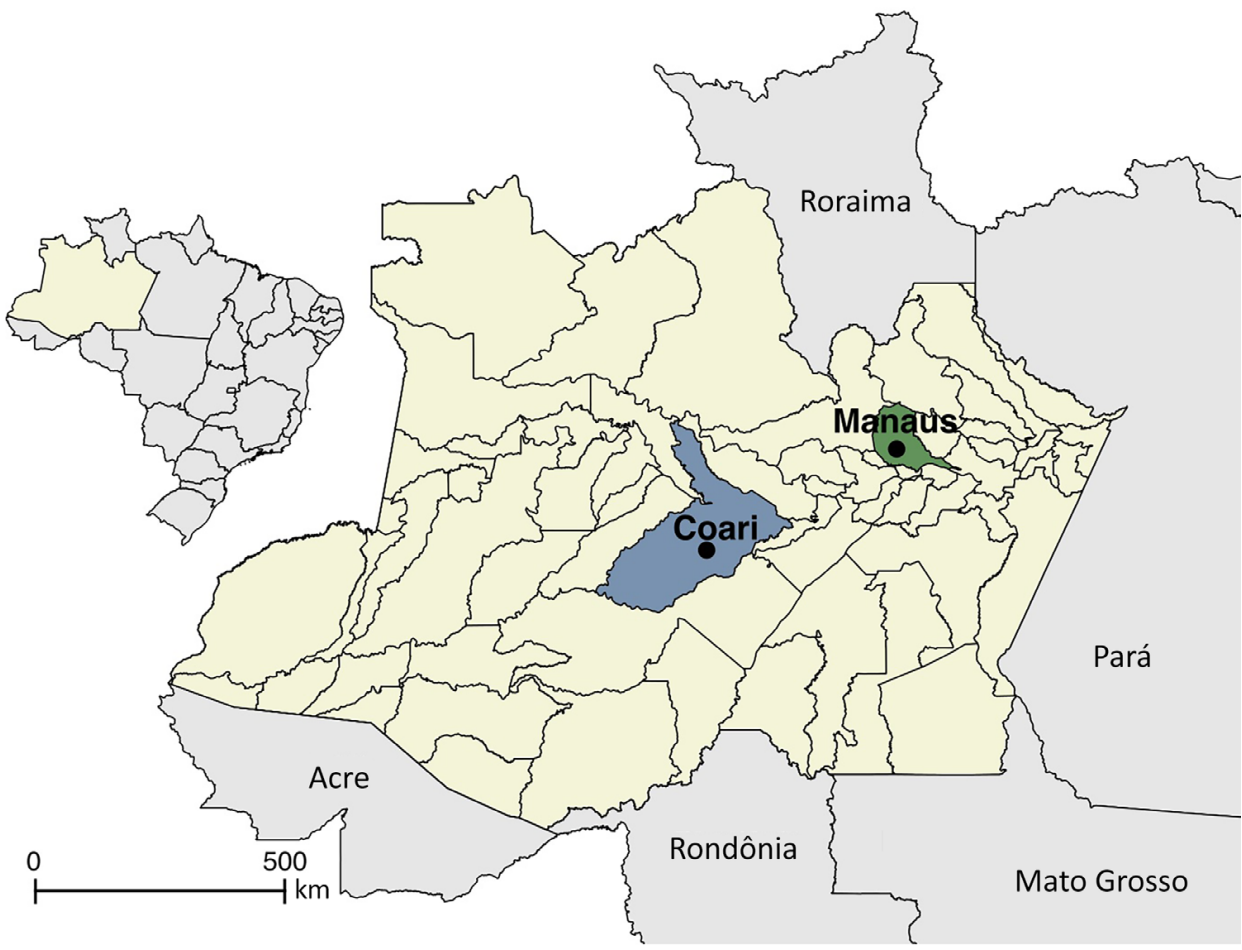
Coari and Manaus municipalities within Amazonas, Brazil, in seroepidemiologic study of Oropouche virus, 2015–2016. Inset shows location of Amazonas state in Brazil.

We analyzed serum samples from 814 persons, 374 (45.9%) participants residing in Coari and 440 (54.1%) in Manaus. Median age was 36 (interquartile range [IQR] 26–48) years; 567 (69.7%) participants were female and 247 (30.3%) male. We detected OROV neutralizing antibodies in 85 (10.4%, 95% CI 8.5%–12.8%) participants (Figure 2, panel A). Seroprevalence, as indicated by presence of neutralizing antibodies, was higher in Manaus (49/440 [11.1%, 95% CI 8.4%–14.5%] positive) than in Coari (9.6%, 36/374, 95% CI 6.9%–13.2%) (Figure 2, panel A). The median PRNT_90_ titer in Manaus was 80 (IQR 40–320), whereas in Coari the median PRNT_90_ titer was 80 (IQR 20–160) (Figure 2, panel B). Participants testing seropositive for OROV had a median age of 47 (IQR 38–54) years; median age in Manaus was 48 (IQR 42–54) years and in Coari, 45 (IQR 33–53) years. OROV seropositivity rates were higher in female than male participants (65/85 [76.5%]) overall, 39/49 (79.6%) in Manaus and 26/36 (72.2%) in Coari. We observed no statistically significant difference in sex or age distribution in neutralizing antibody–positive participants between Coari and Manaus.

**Figure 2:**
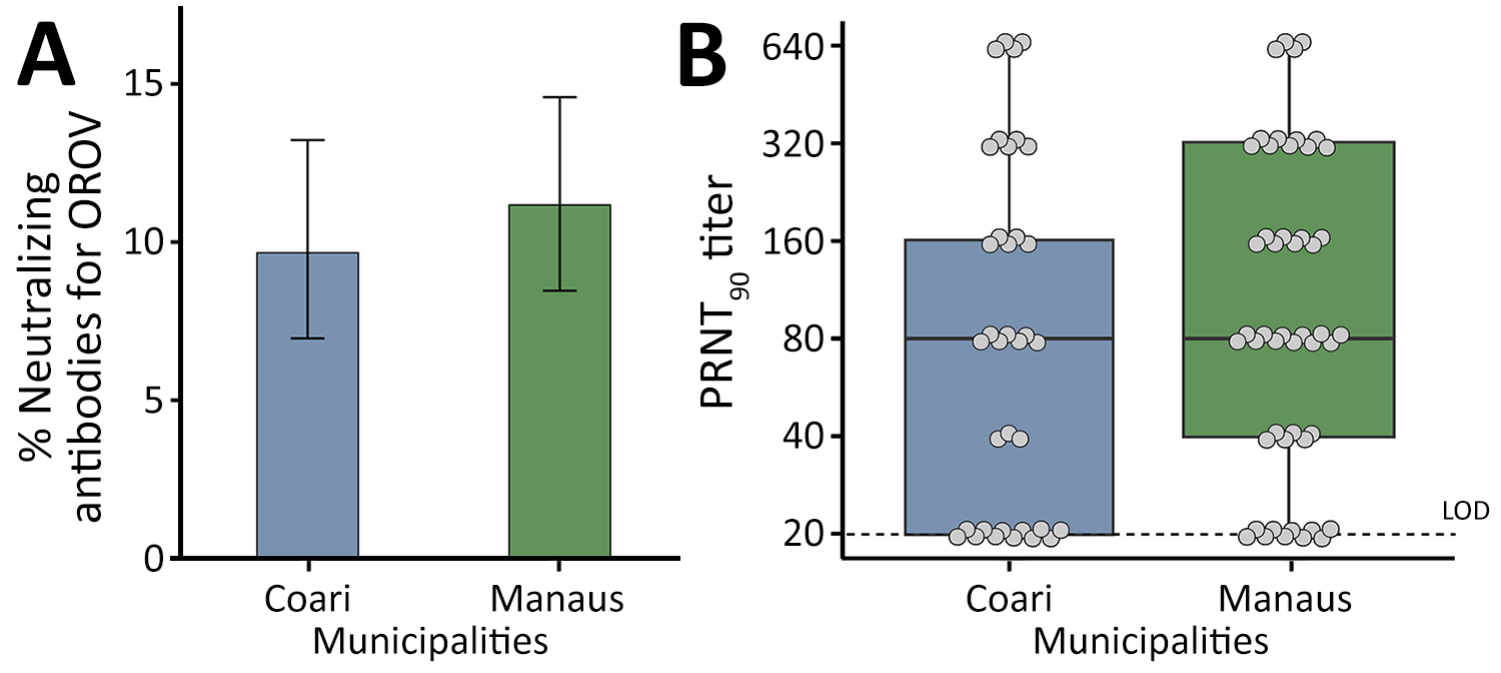
Serosurvey of Oropouche virus in Coari and Manaus municipalities, Amazonas state, Brazil, 2015–2016. A) Percentage of serum samples positive for neutralizing antibodies for OROV per municipality. Error bars indicate 95% CIs. B) Box plot showing the distribution of PRNT_90_ titers of serum samples by municipality. Solid black horizontal line represents the median, box upper and lower limits represent the 75th and 25th percentiles, and whiskers represent minimum and maximum values. Dashed line represents LOD. Dots represent individual healthy volunteers. LOD, limit of detection; NAb, neutralizing antibodies; OROV, Oropouche virus; PRNT_90_, 90% plaque reduction neutralization test.

Our findings identified OROV neutralizing antibodies in residents of Coari and Manaus, suggesting prior OROV transmission in those locations during 2015–2016. The seropositivity rate found in our study was lower than that reported in previous studies from the Peruvian Amazon (18%–35%) (*6*,*7*). However, our results are similar to those of a 2014–2015 study in Amapá State, Brazil, which found a 10.2% seropositivity rate (*8*). We cannot ascertain the timing of OROV infection on the basis of neutralizing antibody data; however, some seropositive cases might be linked with OROV outbreak in Manaus during 1980–1981, where 83/496 (16.7%) of participants from 6 neighborhoods in Manaus tested positive for OROV-specific antibodies after a major OROV outbreak (*9*). Conversely, Oropouche fever was first reported to the Ministry of Health in Coari on December 18, 2023, suggesting a potential undetected OROV transmission not captured by the local surveillance system. We hypothesize that underreporting of OROV infections in Coari could result from several factors, including variable healthcare-seeking behavior, potential higher levels of oligosymptomatic or asymptomatic cases during OROV infection, and misdiagnosis from similar clinical manifestations of other endemic arboviruses, such as dengue, chikungunya, Zika, and Mayaro viruses (*10*).

A limitation of our study is that we focused on previous OROV infections, using a serologic approach for previous infection; molecular studies and detection of IgM are needed to determine the percentage of participants actively infected, particularly during outbreaks. Second, we were unable to determine whether OROV infections occurred in urban or forest settings. Third, some positive samples could have resulted from the cross-reaction with other orthobunyaviruses that remain to be detected in this region. Finally, larger-scale longitudinal studies are needed to determine the effect of OROV, both before and after future OROV outbreaks.

In conclusion, our study demonstrated evidence of OROV circulation in Coari and Manaus before 2015–2016. Our findings highlight the critical need for continuous molecular and serologic laboratory surveillance for OROV to accurately assess its burden in the Amazon Region and beyond, particularly since its reemergence in 2023.

Appendix Additional information about a seroepidemiologic study of Oropouche virus, Amazonas State, Brazil, 2015–2016.
