# Supplementary material for: Seroepidemiologic Study of Oropouche Virus, Amazonas State, Brazil, 2015–2016
Source: Emerg Infect Dis. 2026 Apr;32(4):663–5. doi: 10.3201/eid3204.250917 (PMC13094830; doi:10.3201/eid3204.250917)
Supplement: Appendix — Additional information about a seroepidemiologic study of Oropouche virus, Amazonas State, Brazil, 2015–2016. [file 25-0917-Techapp-s1.pdf]

Article DOI: https://doi.org/10.3201/eid3204.250917

EID cannot ensure accessibility for supplementary materials supplied by authors. Readers who have difficulty accessing supplementary content should contact the authors for assistance.

Seroepidemiologic Study of Oropouche Virus, Amazonas State, Brazil, 2015–2016

Appendix

Materials and Methods

Human Blood Samples Collection

Blood samples from individuals were obtained by venipuncture between 2015 and 2016 from residents of Manaus and Coari municipalities, Amazonas State, Brazil. No participants reported any symptoms at the time of the blood sample collection. Upon arrival at the laboratory, blood samples were centrifuged at 3,000 rpm for 15 minutes to separate the serum, and then the serum samples were stored at −80°C. Samples from Coari and Manaus were partially included in previous studies (*1,2*).

Focus Reduction Neutralization Test for Oropouche Virus

A focus reduction neutralization test was performed to detect the presence of neutralizing antibodies against Oropouche virus (OROV) in human serum samples. Briefly, the samples were diluted in a proportion of 1/10 in Minimum Essential Eagle's Medium (DMEM) and incubated with a solution containing 2×10^3 PFU/mL of OROV BeAn 19991 isolate for 1 hour at 37°C. Next, 100 µL of the virus-serum mixtures were transferred to 96-well plates containing Vero CCL-81 cells (5×10^4 cells per well) with 80% confluence, which were incubated for 1 hour at 37°C with 5% CO_2 for viral adsorption. Subsequently, 125 µL of DMEM containing 0.75% carboxymethylcellulose and 5% fetal bovine serum (FBS) was added to the wells, and the plates were incubated at 37°C with 5% CO_2 for 48 hours. Next, the cells were fixed with 70 µL of 8% paraformaldehyde solution (PFA) and incubated for 1 hour at 4ºC. After removing the PFA, the cells were washed with phosphate-buffered saline (PBS). The cell monolayer was then blocked for 30 minutes with 150 µL of blotto. After blocking, the monolayers were washed with Perm/Wash Buffer (PBS supplemented with 0.1% bovine serum albumin and 0.1% Triton X-

100) and incubated with the polyclonal anti-OROV antibody (Cat no. VR-1228AF, ATTC, USA). After a second wash with Perm/Wash, the monolayers were incubated with an anti-mouse IgG secondary antibody (Cat no. AP124P, Sigma-Aldrich, USA). Finally, after a final wash with Perm/Wash Buffer, the assay was revealed using the True-Blue Peroxidase substrate (Cat no. 5510-0030, KPL, USA) for 30 minutes. OROV neutralizing serum samples were defined as those that promoted at least a 50% reduction in focus formation compared to positive control.

Plaque Reduction Neutralization Test for Oropouche Virus

To assess the titer of neutralizing antibodies against OROV in previous positive OROV neutralizing serum samples, we performed a plaque reduction neutralization test value 90 ($PRNT_{90}$) as described elsewhere (*3*). Briefly, we inactivated the complement system by heating serum samples at 56ºC degrees, then we performed serial dilutions of each serum sample and incubated with a solution containing 2×10^3 PFU/mL of OROV BeAn 19991 isolate for 1 hour at 37°C. Subsequently, the virus-serum mixtures were added to pre-formed Vero CCL-81 cell monolayers and incubated for 1 hour at 37°C in a 5% CO_2 atmosphere. Next, we removed the inoculum and 1 mL of DMEM containing 0.75% carboxymethylcellulose and 5% FBS was gently added to each well, and the plates were incubated at 37°C in a 5% CO_2 atmosphere for 3 days. Finally, the cells were fixed with 500 µL of 8% paraformaldehyde solution for 1 hour and stained with 1% methylene blue (Cat no. PHR3838, Sigma-Aldrich, USA) for 5 minutes. Plaque reduction was calculated as the average of values from two technical duplicates, corresponding to the percentages of the number of plaques counted compared to the positive control. These values were transformed to Log10 for better visualization in the graph and subjected to a three-parameter nonlinear dose response inhibition regression test (Appendix Figure).

Statistical Analysis

Descriptive statistics were conducted in RStudio, version 2024.12.0+467. The R packages required were *readxl*, *ggplot2*, *dplyr*, and *scales*. Sex and age groups were compared using a two-way ANOVA, followed by Tukey's honest significant difference test. For analysis of neutralizing antibody titers, the mean $PRNT_{90}$ for each sample was calculated as the mean of two technical duplicates relative to the positive control using a nonlinear regression curve in GraphPad Prism, version 8.2.1.

References

1. Scachetti GC, Forato J, Claro IM, Hua X, Salgado BB, Vieira A, et al. Re-emergence of Oropouche virus between 2023 and 2024 in Brazil: an observational epidemiological study. Lancet Infect Dis. 2025;25:166–75. PubMed https://doi.org/10.1016/S1473-3099(24)00619-4

2. Manuli ER, Hua X, Scachetti GC, Forato J, Claro IM, Pereira GM, et al. Transmission dynamics of Oropouche virus in Latin America and the Caribbean. Nat Med. 2026. In press. PubMed https://doi.org/10.1038/s41591-026-04221-z

3. Proenca-Modena JL, Hyde JL, Sesti-Costa R, Lucas T, Pinto AK, Richner JM, et al. Interferon-regulatory factor 5-dependent signaling restricts orthobunyavirus dissemination to the central nervous system. J Virol. 2016;90:189–205. PubMed https://doi.org/10.1128/JVI.02276-15

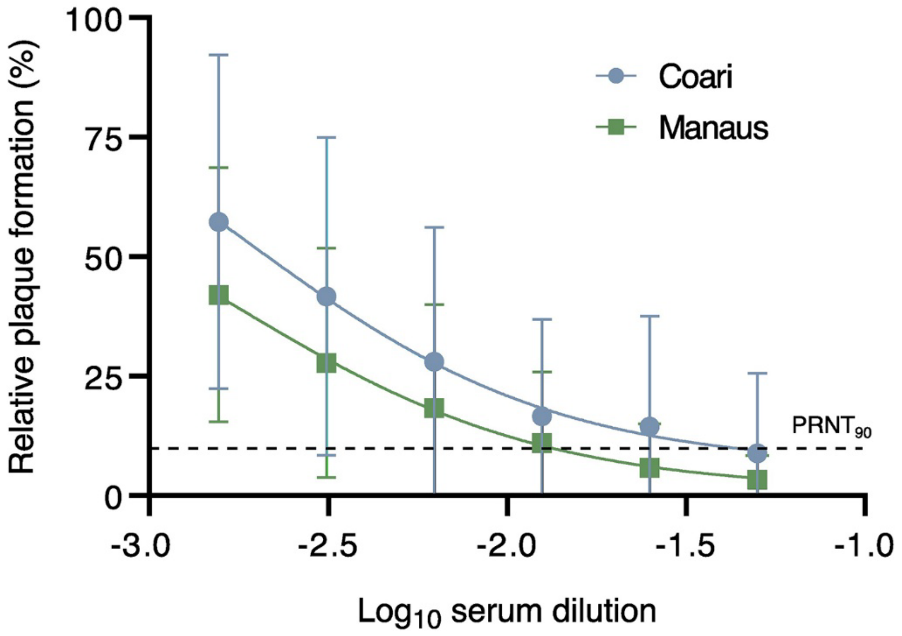

Appendix Table. Data from serosurvey of Oropouche virus in Coari and Manaus, Amazonas State, Brazil, 2015–2016.

ID	Date	Age (years)	Sex	Municipality	$PRNT_{90}$ titer
H033	2015	38	M	Manaus	Below LOD
H034	2015	27	M	Manaus	Below LOD
H036	2015	40	F	Manaus	Below LOD
H038	2015	49	M	Manaus	Below LOD
H039	2015	57	M	Manaus	Below LOD
H040	2015	31	M	Manaus	Below LOD
H041	2015	34	F	Manaus	Below LOD
H042	2015	33	F	Manaus	Below LOD
H044	2015	33	F	Manaus	Below LOD
H048	2015	53	F	Manaus	Below LOD
H049	2015	61	F	Manaus	Below LOD
H050	2015	19	F	Manaus	Below LOD
H051	2015	24	F	Manaus	Below LOD
H052	2015	63	F	Manaus	Below LOD
H054	2015	41	F	Manaus	Below LOD
H055	2015	25	F	Manaus	Below LOD

ID	Date	Age (years)	Sex	Municipality	$PRNT_{90}$ titer
H056	2015	20	F	Manaus	Below LOD
H057	2015	42	F	Manaus	Below LOD
H059	2015	41	F	Manaus	1/320
H060	2015	54	F	Manaus	Below LOD
H061	2015	31	F	Manaus	Below LOD
H062	2015	40	F	Manaus	Below LOD
H063	2015	36	F	Manaus	Below LOD
H064	2015	46	M	Manaus	Below LOD
H066	2015	60	M	Manaus	Below LOD
H067	2015	17	F	Manaus	Below LOD
H068	2015	37	F	Manaus	Below LOD
H070	2015	62	F	Manaus	Below LOD
H071	2015	18	F	Manaus	Below LOD
H072	2015	22	F	Manaus	Below LOD
H073	2015	36	M	Manaus	Below LOD
H074	2015	57	F	Manaus	1/320
H075	2015	28	F	Manaus	Below LOD
H076	2015	19	F	Manaus	Below LOD
H077	2015	44	M	Manaus	Below LOD
H078	2015	37	F	Manaus	Below LOD
H079	2015	65	F	Manaus	Below LOD
H080	2015	22	F	Manaus	Below LOD
H081	2015	47	F	Manaus	1/80
H082	2015	34	F	Manaus	Below LOD
H083	2015	32	M	Manaus	Below LOD
H084	2015	26	M	Manaus	Below LOD
H085	2015	42	F	Manaus	Below LOD
H086	2015	28	M	Manaus	Below LOD
H087	2015	33	M	Manaus	Below LOD
H088	2015	26	M	Manaus	Below LOD
H089	2015	45	F	Manaus	Below LOD
H090	2015	39	M	Manaus	Below LOD
H091	2015	47	F	Manaus	Below LOD
H092	2015	33	M	Manaus	Below LOD
H093	2015	31	F	Manaus	Below LOD
H094	2015	23	F	Manaus	Below LOD
H095	2015	54	M	Manaus	Below LOD
H096	2015	53	F	Manaus	Below LOD
H097	2015	39	F	Manaus	Below LOD
H098	2015	33	F	Manaus	Below LOD
H099	2015	34	M	Manaus	Below LOD
H100	2015	77	F	Manaus	Below LOD
H101	2015	NA	M	Manaus	Below LOD
H102	2015	NA	F	Manaus	Below LOD
H103	2015	32	M	Manaus	Below LOD
H104	2015	NA	F	Manaus	1/160
H105	2015	NA	M	Manaus	Below LOD
H106	2015	NA	F	Manaus	Below LOD
H107	2015	NA	F	Manaus	1/160
H108	2015	NA	F	Manaus	Below LOD
H109	2015	NA	F	Manaus	1/80
H110	2015	NA	F	Manaus	Below LOD
H111	2015	NA	F	Manaus	Below LOD
H112	2015	NA	F	Manaus	Below LOD
H113	2015	NA	M	Manaus	Below LOD
H114	2015	NA	F	Manaus	1/80
H115	2015	NA	F	Manaus	Below LOD
H116	2015	NA	F	Manaus	Below LOD
H117	2015	NA	F	Manaus	Below LOD
H118	2015	NA	M	Manaus	Below LOD
H119	2015	18	F	Manaus	Below LOD
H120	2015	17	F	Manaus	Below LOD
H121	2015	NA	F	Manaus	Below LOD
H122	2015	19	M	Manaus	Below LOD
H123	2015	NA	M	Manaus	Below LOD
H124	2015	NA	M	Manaus	Below LOD
H125	2015	NA	M	Manaus	Below LOD
H126	2015	NA	F	Manaus	Below LOD
H127	2015	NA	F	Manaus	Below LOD

ID	Date	Age (years)	Sex	Municipality	$PRNT_{90}$ titer
H128	2015	19	F	Manaus	Below LOD
H129	2015	NA	F	Manaus	Below LOD
H130	2015	NA	M	Manaus	Below LOD
H131	2015	NA	M	Manaus	Below LOD
H132	2015	NA	M	Manaus	Below LOD
H133	2015	NA	F	Manaus	Below LOD
H134	2015	NA	F	Manaus	1/160
H135	2015	NA	M	Manaus	Below LOD
H136	2015	NA	F	Manaus	Below LOD
H137	2015	NA	M	Manaus	Below LOD
H138	2015	NA	M	Manaus	Below LOD
H139	2015	NA	F	Manaus	Below LOD
H140	2015	NA	F	Manaus	Below LOD
H141	2015	NA	F	Manaus	Below LOD
H142	2015	NA	M	Manaus	Below LOD
H143	2015	NA	F	Manaus	Below LOD
H144	2015	NA	F	Manaus	Below LOD
H145	2015	NA	F	Manaus	Below LOD
H146	2015	NA	M	Manaus	Below LOD
H147	2015	NA	M	Manaus	Below LOD
H148	2015	NA	M	Manaus	Below LOD
H149	2015	NA	F	Manaus	Below LOD
H150	2015	NA	F	Manaus	Below LOD
H151	2015	NA	F	Manaus	Below LOD
H152	2015	NA	M	Manaus	Below LOD
H153	2015	NA	F	Manaus	Below LOD
H154	2015	NA	F	Manaus	Below LOD
H155	2015	NA	F	Manaus	Below LOD
H157	2015	NA	F	Manaus	Below LOD
H158	2015	NA	F	Manaus	Below LOD
H159	2015	NA	M	Manaus	Below LOD
H160	2015	NA	F	Manaus	Below LOD
H161	2015	NA	F	Manaus	Below LOD
H162	2015	NA	F	Manaus	Below LOD
H163	2015	NA	F	Manaus	1/20
H164	2015	NA	M	Manaus	Below LOD
H165	2015	NA	F	Manaus	Below LOD
H166	2015	NA	F	Manaus	1/20
H168	2015	NA	F	Manaus	Below LOD
H175	2015	NA	F	Manaus	Below LOD
H176	2015	NA	M	Manaus	Below LOD
H177	2015	NA	M	Manaus	Below LOD
H178	2015	NA	M	Manaus	Below LOD
H179	2015	NA	F	Manaus	Below LOD
H181	2015	NA	F	Manaus	Below LOD
H183	2015	NA	F	Manaus	Below LOD
H184	2015	19	M	Manaus	Below LOD
H185	2015	NA	M	Manaus	Below LOD
H186	2015	NA	M	Manaus	1/20
H187	2015	NA	F	Manaus	Below LOD
H188	2015	NA	F	Manaus	Below LOD
H189	2015	NA	M	Manaus	Below LOD
H191	2015	35	M	Manaus	Below LOD
H192	2015	NA	F	Manaus	Below LOD
H193	2015	22	M	Manaus	Below LOD
H194	2015	18	F	Manaus	Below LOD
H197	2015	NA	F	Manaus	Below LOD
H199	2015	22	M	Manaus	Below LOD
H201	2015	25	M	Manaus	Below LOD
H203	2015	33	F	Manaus	Below LOD
H204	2015	27	F	Manaus	Below LOD
H205	2015	35	F	Manaus	Below LOD
H207	2015	29	M	Manaus	Below LOD
H208	2015	62	F	Manaus	1/40
H213	2015	50	F	Manaus	Below LOD
H215	2015	34	F	Manaus	Below LOD
H216	2015	39	M	Manaus	Below LOD
H219	2015	NA	F	Manaus	Below LOD
H220	2015	NA	F	Manaus	Below LOD

ID	Date	Age (years)	Sex	Municipality	$PRNT_{90}$ titer
H221	2015	NA	M	Manaus	1/640
H222	2015	48	F	Manaus	1/20
H223	2015	70	F	Manaus	Below LOD
H224	2015	26	F	Manaus	Below LOD
H225	2015	23	F	Manaus	Below LOD
H227	2015	NA	F	Manaus	Below LOD
H229	2015	NA	M	Manaus	Below LOD
H230	2015	NA	F	Manaus	Below LOD
H231	2015	NA	F	Manaus	Below LOD
H233	2015	NA	F	Manaus	Below LOD
H234	2015	19	F	Manaus	Below LOD
H235	2015	NA	F	Manaus	Below LOD
H236	2015	NA	F	Manaus	Below LOD
H237	2015	45	F	Manaus	Below LOD
H238	2015	15	M	Manaus	Below LOD
H240	2015	41	F	Manaus	Below LOD
H241	2015	52	M	Manaus	Below LOD
H242	2015	24	M	Manaus	Below LOD
H243	2015	36	F	Manaus	Below LOD
H244	2015	51	F	Manaus	Below LOD
H252	2015	18	F	Manaus	Below LOD
H254	2015	23	F	Manaus	Below LOD
H255	2015	25	F	Manaus	Below LOD
H256	2015	69	M	Manaus	1/160
H257	2015	17	M	Manaus	Below LOD
H258	2015	44	F	Manaus	Below LOD
H259	2015	23	M	Manaus	Below LOD
H260	2015	48	F	Manaus	1/20
H261	2015	38	F	Manaus	Below LOD
H262	2015	43	F	Manaus	1/20
H264	2015	19	F	Manaus	Below LOD
H265	2015	34	F	Manaus	Below LOD
H267	2015	45	F	Manaus	Below LOD
H268	2015	27	F	Manaus	Below LOD
H269	2015	41	M	Manaus	Below LOD
H272	2015	48	F	Manaus	Below LOD
H273	2015	45	F	Manaus	Below LOD
H274	2015	18	M	Manaus	Below LOD
H275	2015	42	F	Manaus	Below LOD
H276	2015	37	M	Manaus	Below LOD
H277	2015	33	F	Manaus	Below LOD
H278	2015	39	F	Manaus	Below LOD
H279	2015	40	F	Manaus	Below LOD
H280	2015	29	F	Manaus	Below LOD
H281	2015	35	M	Manaus	Below LOD
H282	2015	53	M	Manaus	Below LOD
H283	2015	43	F	Manaus	Below LOD
H284	2015	43	F	Manaus	Below LOD
H285	2015	34	M	Manaus	Below LOD
H286	2015	19	F	Manaus	Below LOD
H287	2015	56	F	Manaus	Below LOD
H288	2015	16	F	Manaus	Below LOD
H289	2015	64	M	Manaus	Below LOD
H291	2015	76	M	Manaus	1/40
H292	2015	27	F	Manaus	Below LOD
H293	2015	59	M	Manaus	Below LOD
H296	2015	51	F	Manaus	1/640
H297	2015	47	F	Manaus	Below LOD
H299	2015	55	F	Manaus	1/80
H300	2015	24	F	Manaus	Below LOD
H301	2015	36	F	Manaus	Below LOD
H303	2015	35	F	Manaus	Below LOD
H304	2015	31	F	Manaus	Below LOD
H305	2015	NA	F	Manaus	Below LOD
H306	2015	NA	F	Manaus	Below LOD
H307	2015	NA	F	Manaus	Below LOD
H308	2015	NA	F	Manaus	Below LOD
H309	2015	NA	F	Manaus	Below LOD
H310	2015	NA	F	Manaus	Below LOD

ID	Date	Age (years)	Sex	Municipality	$PRNT_{90}$ titer
H311	2015	NA	F	Manaus	Below LOD
H312	2015	NA	F	Manaus	Below LOD
H313	2015	NA	F	Manaus	Below LOD
H314	2015	NA	M	Manaus	Below LOD
H315	2015	NA	F	Manaus	Below LOD
H316	2015	NA	F	Manaus	Below LOD
H317	2015	57	F	Manaus	Below LOD
H318	2015	54	F	Manaus	1/320
H319	2015	58	F	Manaus	Below LOD
H321	2015	32	M	Manaus	Below LOD
H322	2015	53	F	Manaus	Below LOD
H324	2015	44	F	Manaus	Below LOD
H325	2015	65	F	Manaus	Below LOD
H326	2015	57	F	Manaus	Below LOD
H327	2015	66	M	Manaus	Below LOD
H329	2015	44	F	Manaus	Below LOD
H330	2015	74	M	Manaus	Below LOD
H332	2015	18	F	Manaus	Below LOD
H333	2015	27	F	Manaus	Below LOD
H334	2015	32	F	Manaus	Below LOD
H335	2015	17	F	Manaus	Below LOD
H336	2015	24	F	Manaus	Below LOD
H339	2015	21	M	Manaus	Below LOD
H340	2015	53	F	Manaus	1/640
H341	2015	57	M	Manaus	Below LOD
H343	2015	24	M	Manaus	Below LOD
H344	2015	55	M	Manaus	Below LOD
H345	2015	58	M	Manaus	Below LOD
H346	2015	21	F	Manaus	Below LOD
H347	2015	40	F	Manaus	Below LOD
H348	2015	47	F	Manaus	1/80
H349	2015	53	M	Manaus	Below LOD
H350	2015	68	F	Manaus	1/80
H351	2015	41	F	Manaus	1/160
H352	2015	46	F	Manaus	Below LOD
H354	2015	46	F	Manaus	Below LOD
H355	2015	34	M	Manaus	Below LOD
H356	2015	36	F	Manaus	1/20
H357	2015	27	F	Manaus	Below LOD
H358	2015	43	M	Manaus	1/20
H359	2015	43	F	Manaus	Below LOD
H361	2015	48	F	Manaus	Below LOD
H362	2015	56	M	Manaus	Below LOD
H363	2015	27	F	Manaus	Below LOD
H364	2015	20	F	Manaus	Below LOD
H368	2015	23	M	Manaus	Below LOD
H369	2015	31	F	Manaus	Below LOD
H370	2015	47	M	Manaus	1/640
H371	2015	40	F	Manaus	Below LOD
H372	2015	28	F	Manaus	Below LOD
H373	2015	68	F	Manaus	1/640
H374	2015	50	F	Manaus	Below LOD
H375	2015	37	F	Manaus	Below LOD
H376	2015	34	M	Manaus	Below LOD
H378	2015	32	F	Manaus	Below LOD
H379	2015	47	F	Manaus	Below LOD
H380	2015	38	F	Manaus	Below LOD
H381	2015	32	F	Manaus	1/320
H382	2015	62	F	Manaus	Below LOD
H383	2015	31	F	Manaus	Below LOD
H391	2015	29	F	Manaus	Below LOD
H392	2015	48	M	Manaus	Below LOD
H394	2015	62	F	Manaus	Below LOD
H396	2015	44	F	Manaus	Below LOD
H397	2015	21	F	Manaus	Below LOD
H398	2015	42	M	Manaus	1/320
H399	2015	52	M	Manaus	Below LOD
H400	2015	27	F	Manaus	Below LOD
H403	2015	38	M	Manaus	Below LOD

ID	Date	Age (years)	Sex	Municipality	$PRNT_{90}$ titer
H404	2015	39	F	Manaus	1/80
H405	2015	20	F	Manaus	Below LOD
H415	2015	20	F	Manaus	Below LOD
H416	2015	38	F	Manaus	Below LOD
H417	2015	45	F	Manaus	Below LOD
H419	2015	43	F	Manaus	Below LOD
H420	2015	62	F	Manaus	Below LOD
H421	2015	53	F	Manaus	Below LOD
H422	2015	30	F	Manaus	Below LOD
H424	2015	57	M	Manaus	Below LOD
H425	2015	39	M	Manaus	Below LOD
H426	2015	44	F	Manaus	Below LOD
H427	2015	54	F	Manaus	1/320
H428	2015	44	F	Manaus	1/20
H429	2015	45	F	Manaus	Below LOD
H430	2015	51	M	Manaus	Below LOD
H436	2015	53	F	Manaus	1/40
H437	2015	33	F	Manaus	Below LOD
H438	2015	45	F	Manaus	Below LOD
H439	2015	25	F	Manaus	Below LOD
H440	2015	18	M	Manaus	Below LOD
H441	2015	24	M	Manaus	Below LOD
H442	2015	14	F	Manaus	Below LOD
H443	2015	26	F	Manaus	Below LOD
H444	2015	42	F	Manaus	Below LOD
H445	2015	30	M	Manaus	Below LOD
H446	2015	19	F	Manaus	Below LOD
H447	2015	63	F	Manaus	Below LOD
H448	2015	18	F	Manaus	Below LOD
H449	2015	44	M	Manaus	Below LOD
H450	2015	29	F	Manaus	Below LOD
H451	2015	21	F	Manaus	Below LOD
H452	2015	31	F	Manaus	Below LOD
H453	2015	62	M	Manaus	Below LOD
H454	2015	63	F	Manaus	Below LOD
H458	2015	34	F	Manaus	Below LOD
H460	2015	39	M	Manaus	Below LOD
H461	2015	54	F	Manaus	Below LOD
H462	2015	25	F	Manaus	Below LOD
H463	2015	17	F	Manaus	Below LOD
H464	2015	29	M	Manaus	Below LOD
H466	2015	44	F	Manaus	1/320
H467	2015	NA	M	Manaus	Below LOD
H468	2015	41	F	Manaus	Below LOD
H469	2015	41	F	Manaus	Below LOD
H470	2015	52	F	Manaus	1/160
H471	2015	22	F	Manaus	Below LOD
H472	2015	51	M	Manaus	Below LOD
H473	2015	25	M	Manaus	Below LOD
H474	2015	23	F	Manaus	Below LOD
H475	2015	NA	M	Manaus	Below LOD
H476	2015	20	M	Manaus	Below LOD
H477	2015	38	F	Manaus	Below LOD
H478	2015	37	F	Manaus	Below LOD
H479	2015	36	F	Manaus	Below LOD
H480	2015	18	M	Manaus	Below LOD
H482	2015	27	F	Manaus	Below LOD
H484	2015	54	M	Manaus	Below LOD
H485	2015	34	M	Manaus	Below LOD
H486	2015	NA	M	Manaus	1/80
H487	2015	51	F	Manaus	1/320
H488	2015	55	M	Manaus	Below LOD
H489	2015	38	F	Manaus	Below LOD
H500	2015	62	F	Manaus	Below LOD
H501	2015	38	F	Manaus	Below LOD
H502	2015	75	M	Manaus	Below LOD
H503	2015	54	F	Manaus	Below LOD
H504	2015	52	F	Manaus	Below LOD
H505	2015	23	F	Manaus	Below LOD

ID	Date	Age (years)	Sex	Municipality	$PRNT_{90}$ titer
H506	2015	25	F	Manaus	Below LOD
H507	2015	45	F	Manaus	Below LOD
H508	2015	29	F	Manaus	Below LOD
H509	2015	33	F	Manaus	Below LOD
H510	2015	25	M	Manaus	Below LOD
H511	2015	49	F	Manaus	1/640
H512	2015	59	F	Manaus	Below LOD
H513	2015	38	F	Manaus	1/40
H514	2015	51	F	Manaus	Below LOD
H515	2015	25	F	Manaus	Below LOD
H516	2015	31	F	Manaus	Below LOD
H518	2015	NA	M	Manaus	Below LOD
H519	2015	48	F	Manaus	1/80
H520	2015	18	F	Manaus	1/160
H521	2015	28	F	Manaus	1/160
H522	2015	19	F	Manaus	1/20
H523	2015	32	F	Manaus	Below LOD
H524	2015	60	M	Manaus	Below LOD
H526	2015	52	M	Manaus	1/320
H527	2015	17	F	Manaus	Below LOD
H529	2015	46	F	Manaus	Below LOD
H530	2015	44	F	Manaus	1/40
H531	2015	NA	M	Manaus	Below LOD
H532	2015	54	F	Manaus	Below LOD
H533	2015	35	F	Manaus	Below LOD
H534	2015	43	F	Manaus	Below LOD
H535	2015	50	M	Manaus	Below LOD
H536	2015	26	M	Manaus	Below LOD
H537	2015	26	F	Manaus	Below LOD
H539	2015	48	F	Manaus	Below LOD
H540	2015	44	M	Manaus	Below LOD
H541	2015	45	M	Manaus	Below LOD
H542	2015	31	F	Manaus	Below LOD
H543	2015	17	M	Manaus	Below LOD
H544	2015	23	M	Manaus	Below LOD
H545	2015	32	F	Manaus	Below LOD
H546	2015	33	F	Manaus	Below LOD
H547	2015	59	F	Manaus	Below LOD
H548	2015	30	M	Manaus	Below LOD
H549	2015	39	F	Manaus	Below LOD
H550	2015	33	M	Manaus	Below LOD
H551	2015	NA	F	Manaus	Below LOD
H552	2015	25	M	Manaus	Below LOD
H553	2015	37	F	Manaus	Below LOD
H554	2015	46	F	Manaus	Below LOD
H555	2015	19	F	Manaus	Below LOD
H556	2015	53	F	Manaus	Below LOD
H557	2015	24	F	Manaus	Below LOD
H558	2015	23	F	Manaus	Below LOD
H559	2015	41	M	Manaus	Below LOD
H560	2015	37	F	Manaus	Below LOD
H561	2015	NA	M	Manaus	Below LOD
H562	2015	20	F	Manaus	Below LOD
H563	2015	NA	F	Manaus	Below LOD
H565	2015	NA	F	Manaus	Below LOD
H567	2015	NA	F	Manaus	Below LOD
H568	2015	NA	F	Manaus	Below LOD
H569	2015	NA	F	Manaus	Below LOD
H571	2015	NA	F	Manaus	Below LOD
H572	2015	NA	M	Manaus	Below LOD
H573	2015	NA	F	Manaus	Below LOD
H574	2015	NA	F	Manaus	Below LOD
H575	2015	NA	F	Manaus	Below LOD
H576	2015	NA	F	Manaus	Below LOD
H577	2015	NA	F	Manaus	Below LOD
H578	2015	57	F	Manaus	1/40
H579	2015	NA	M	Manaus	1/160
H580	2015	18	F	Manaus	Below LOD
H583	2015	NA	F	Manaus	Below LOD

ID	Date	Age (years)	Sex	Municipality	$PRNT_{90}$ titer
H584	2015	67	M	Manaus	Below LOD
H585	2015	NA	M	Manaus	Below LOD
H586	2015	19	F	Manaus	Below LOD
H588	2015	NA	F	Manaus	Below LOD
H589	2015	NA	F	Manaus	Below LOD
H590	2015	NA	F	Manaus	Below LOD
H591	2015	NA	M	Manaus	Below LOD
H592	2015	NA	M	Manaus	Below LOD
H595	2015	NA	F	Manaus	Below LOD
H597	2015	NA	F	Manaus	Below LOD
A8	2016	29	M	Coari	Below LOD
A9	2016	25	F	Coari	Below LOD
A10	2016	19	F	Coari	Below LOD
A11	2016	61	F	Coari	1/20
A12	2016	41	M	Coari	Below LOD
A13	2016	26	F	Coari	Below LOD
A14	2016	34	M	Coari	Below LOD
A15	2016	19	M	Coari	Below LOD
A16	2016	26	F	Coari	Below LOD
A17	2016	54	M	Coari	1/20
A18	2016	46	F	Coari	Below LOD
A19	2016	45	M	Coari	1/160
A20	2016	19	M	Coari	1/640
A21	2016	23	M	Coari	1/20
A23	2016	33	M	Coari	1/20
A24	2016	38	F	Coari	1/20
A25	2016	41	F	Coari	Below LOD
A26	2016	20	M	Coari	Below LOD
A27	2016	22	M	Coari	1/20
A28	2016	59	F	Coari	Below LOD
A29	2016	25	F	Coari	Below LOD
A30	2016	46	F	Coari	1/20
A31	2016	27	F	Coari	Below LOD
A32	2016	47	F	Coari	1/20
A33	2016	19	F	Coari	1/320
A34	2016	24	F	Coari	1/20
A35	2016	66	F	Coari	1/80
A36	2016	18	F	Coari	Below LOD
A37	2016	76	F	Coari	Below LOD
A38	2016	20	F	Coari	Below LOD
A39	2016	19	F	Coari	Below LOD
A40	2016	60	M	Coari	Below LOD
A41	2016	23	F	Coari	Below LOD
A44	2016	19	F	Coari	Below LOD
A45	2016	58	M	Coari	Below LOD
A46	2016	58	M	Coari	Below LOD
A47	2016	19	F	Coari	Below LOD
A48	2016	57	F	Coari	Below LOD
A50	2016	28	F	Coari	Below LOD
A51	2016	37	F	Coari	Below LOD
A52	2016	56	M	Coari	Below LOD
A53	2016	38	F	Coari	Below LOD
A54	2016	22	F	Coari	Below LOD
A56	2016	22	F	Coari	Below LOD
A57	2016	34	F	Coari	Below LOD
A58	2016	27	M	Coari	Below LOD
A59	2016	37	F	Coari	Below LOD
A60	2016	23	F	Coari	Below LOD
A61	2016	40	F	Coari	Below LOD
A62	2016	33	M	Coari	Below LOD
A63	2016	48	F	Coari	Below LOD
A64	2016	35	F	Coari	Below LOD
A65	2016	48	F	Coari	Below LOD
A66	2016	22	F	Coari	Below LOD
A67	2016	58	M	Coari	Below LOD
A68	2016	30	F	Coari	Below LOD
A69	2016	21	F	Coari	Below LOD
A70	2016	32	F	Coari	Below LOD
A71	2016	24	F	Coari	Below LOD

ID	Date	Age (years)	Sex	Municipality	$PRNT_{90}$ titer
A72	2016	25	F	Coari	Below LOD
A73	2016	58	M	Coari	Below LOD
A74	2016	28	F	Coari	Below LOD
A75	2016	73	M	Coari	Below LOD
A76	2016	32	M	Coari	Below LOD
A77	2016	56	F	Coari	1/80
A78	2016	26	F	Coari	Below LOD
A79	2016	36	M	Coari	Below LOD
A80	2016	68	F	Coari	Below LOD
A81	2016	18	F	Coari	Below LOD
A82	2016	38	F	Coari	Below LOD
A83	2016	38	F	Coari	Below LOD
A84	2016	64	M	Coari	Below LOD
A86	2016	35	M	Coari	Below LOD
A87	2016	37	M	Coari	Below LOD
A88	2016	31	F	Coari	Below LOD
A89	2016	26	F	Coari	Below LOD
A90	2016	19	F	Coari	Below LOD
A91	2016	43	F	Coari	1/320
A92	2016	36	F	Coari	Below LOD
A93	2016	65	F	Coari	Below LOD
A94	2016	84	M	Coari	Below LOD
A95	2016	25	M	Coari	Below LOD
A96	2016	20	F	Coari	Below LOD
A97	2016	21	F	Coari	Below LOD
A98	2016	74	M	Coari	Below LOD
A99	2016	61	F	Coari	Below LOD
A100	2016	37	F	Coari	Below LOD
A101	2016	36	F	Coari	Below LOD
A102	2016	60	F	Coari	Below LOD
A103	2016	23	F	Coari	Below LOD
A104	2016	19	F	Coari	Below LOD
A105	2016	31	F	Coari	Below LOD
A106	2016	38	F	Coari	Below LOD
A107	2016	23	F	Coari	Below LOD
A108	2016	69	M	Coari	Below LOD
A109	2016	46	F	Coari	Below LOD
A110	2016	48	F	Coari	Below LOD
A111	2016	28	F	Coari	1/160
A112	2016	33	F	Coari	Below LOD
A113	2016	38	F	Coari	Below LOD
A114	2016	20	M	Coari	Below LOD
A115	2016	26	F	Coari	Below LOD
A116	2016	22	F	Coari	Below LOD
A117	2016	31	M	Coari	Below LOD
A118	2016	25	M	Coari	Below LOD
A119	2016	21	F	Coari	Below LOD
A120	2016	34	F	Coari	Below LOD
A122	2016	61	M	Coari	Below LOD
A123	2016	58	M	Coari	Below LOD
A125	2016	40	F	Coari	Below LOD
A126	2016	22	F	Coari	Below LOD
A127	2016	65	F	Coari	Below LOD
A128	2016	49	F	Coari	Below LOD
A129	2016	35	F	Coari	Below LOD
A130	2016	40	F	Coari	Below LOD
A131	2016	34	F	Coari	Below LOD
A132	2016	24	M	Coari	Below LOD
A133	2016	40	M	Coari	Below LOD
A134	2016	35	M	Coari	Below LOD
A135	2016	43	M	Coari	Below LOD
A136	2016	33	F	Coari	Below LOD
A137	2016	49	F	Coari	Below LOD
A138	2016	54	F	Coari	Below LOD
A139	2016	29	F	Coari	Below LOD
A140	2016	65	F	Coari	Below LOD
A141	2016	23	M	Coari	Below LOD
A142	2016	20	F	Coari	Below LOD
A143	2016	18	M	Coari	Below LOD

ID	Date	Age (years)	Sex	Municipality	$PRNT_{90}$ titer
A144	2016	43	M	Coari	Below LOD
A145	2016	26	F	Coari	Below LOD
A146	2016	18	F	Coari	Below LOD
A147	2016	29	F	Coari	Below LOD
A148	2016	18	M	Coari	Below LOD
A149	2016	28	F	Coari	Below LOD
A150	2016	38	M	Coari	Below LOD
A151	2016	33	F	Coari	Below LOD
A152	2016	46	F	Coari	Below LOD
A153	2016	27	M	Coari	Below LOD
A154	2016	24	F	Coari	Below LOD
A155	2016	30	F	Coari	Below LOD
A156	2016	27	F	Coari	Below LOD
A157	2016	20	F	Coari	Below LOD
A158	2016	28	F	Coari	Below LOD
A159	2016	36	M	Coari	Below LOD
A160	2016	34	F	Coari	Below LOD
A161	2016	19	F	Coari	Below LOD
A162	2016	58	F	Coari	Below LOD
A163	2016	43	F	Coari	Below LOD
A164	2016	41	M	Coari	Below LOD
A165	2016	48	F	Coari	Below LOD
A166	2016	68	F	Coari	Below LOD
A167	2016	25	F	Coari	Below LOD
A168	2016	24	F	Coari	Below LOD
A169	2016	41	F	Coari	Below LOD
A170	2016	40	F	Coari	Below LOD
A172	2016	27	F	Coari	Below LOD
A173	2016	20	F	Coari	Below LOD
A174	2016	23	F	Coari	Below LOD
A175	2016	21	M	Coari	Below LOD
A176	2016	36	M	Coari	Below LOD
A177	2016	34	F	Coari	Below LOD
A178	2016	19	F	Coari	Below LOD
A179	2016	22	F	Coari	Below LOD
A180	2016	28	F	Coari	Below LOD
A181	2016	56	M	Coari	Below LOD
A182	2016	36	F	Coari	Below LOD
A183	2016	33	F	Coari	Below LOD
A184	2016	25	F	Coari	Below LOD
A186	2016	45	F	Coari	Below LOD
A187	2016	54	F	Coari	Below LOD
A188	2016	57	F	Coari	Below LOD
A189	2016	42	F	Coari	Below LOD
A190	2016	57	F	Coari	Below LOD
A191	2016	53	F	Coari	Below LOD
A192	2016	56	F	Coari	Below LOD
A193	2016	58	F	Coari	1/160
A194	2016	49	F	Coari	Below LOD
A195	2016	60	M	Coari	Below LOD
A196	2016	34	F	Coari	Below LOD
A197	2016	50	F	Coari	Below LOD
A198	2016	54	F	Coari	Below LOD
A199	2016	27	M	Coari	Below LOD
A200	2016	33	F	Coari	1/640
A201	2016	83	M	Coari	Below LOD
A202	2016	21	F	Coari	Below LOD
A203	2016	50	M	Coari	Below LOD
A204	2016	32	F	Coari	Below LOD
A205	2016	41	M	Coari	Below LOD
A206	2016	34	F	Coari	1/320
A207	2016	29	M	Coari	Below LOD
A208	2016	63	M	Coari	Below LOD
A209	2016	18	F	Coari	Below LOD
A210	2016	27	F	Coari	Below LOD
A211	2016	26	F	Coari	Below LOD
A212	2016	33	F	Coari	Below LOD
A213	2016	28	M	Coari	Below LOD
A214	2016	45	F	Coari	Below LOD

ID	Date	Age (years)	Sex	Municipality	$PRNT_{90}$ titer
A215	2016	31	M	Coari	Below LOD
A216	2016	31	F	Coari	1/160
A218	2016	28	F	Coari	Below LOD
A219	2016	42	M	Coari	Below LOD
A220	2016	40	F	Coari	Below LOD
A221	2016	61	M	Coari	Below LOD
A222	2016	50	F	Coari	Below LOD
A223	2016	20	F	Coari	Below LOD
A224	2016	24	M	Coari	Below LOD
A225	2016	39	F	Coari	Below LOD
A226	2016	29	F	Coari	Below LOD
A227	2016	50	F	Coari	Below LOD
A228	2016	29	F	Coari	Below LOD
A229	2016	28	F	Coari	Below LOD
A230	2016	38	F	Coari	Below LOD
A231	2016	34	F	Coari	Below LOD
A233	2016	20	F	Coari	Below LOD
A234	2016	46	M	Coari	Below LOD
A235	2016	43	M	Coari	Below LOD
A237	2016	19	M	Coari	Below LOD
A241	2016	18	M	Coari	Below LOD
A243	2016	54	F	Coari	Below LOD
A244	2016	26	F	Coari	Below LOD
A245	2016	32	F	Coari	Below LOD
A246	2016	22	F	Coari	Below LOD
A247	2016	58	M	Coari	Below LOD
A248	2016	53	F	Coari	Below LOD
A249	2016	18	F	Coari	Below LOD
A250	2016	55	F	Coari	Below LOD
A251	2016	24	F	Coari	Below LOD
A252	2016	22	F	Coari	Below LOD
A253	2016	18	F	Coari	Below LOD
A254	2016	30	F	Coari	Below LOD
A255	2016	45	F	Coari	Below LOD
A256	2016	18	F	Coari	Below LOD
A257	2016	23	F	Coari	Below LOD
A258	2016	31	F	Coari	Below LOD
A259	2016	46	F	Coari	Below LOD
A260	2016	29	F	Coari	Below LOD
A261	2016	41	F	Coari	Below LOD
A262	2016	38	M	Coari	Below LOD
A264	2016	28	M	Coari	Below LOD
A265	2016	27	M	Coari	Below LOD
A266	2016	78	M	Coari	Below LOD
A267	2016	75	M	Coari	Below LOD
A268	2016	41	M	Coari	Below LOD
A269	2016	20	M	Coari	Below LOD
A270	2016	19	M	Coari	Below LOD
A271	2016	46	F	Coari	Below LOD
A272	2016	28	M	Coari	Below LOD
A273	2016	33	M	Coari	Below LOD
A274	2016	44	M	Coari	Below LOD
A275	2016	49	M	Coari	Below LOD
A276	2016	41	M	Coari	Below LOD
A277	2016	32	M	Coari	Below LOD
A278	2016	33	M	Coari	Below LOD
A279	2016	23	F	Coari	Below LOD
A280	2016	41	F	Coari	Below LOD
A281	2016	39	M	Coari	Below LOD
A282	2016	45	F	Coari	Below LOD
A283	2016	48	F	Coari	Below LOD
A284	2016	44	F	Coari	Below LOD
A285	2016	42	F	Coari	Below LOD
A286	2016	38	M	Coari	Below LOD
A287	2016	28	F	Coari	Below LOD
A288	2016	43	F	Coari	Below LOD
A289	2016	56	F	Coari	Below LOD
A290	2016	64	F	Coari	Below LOD
A291	2016	26	F	Coari	1/20

ID	Date	Age (years)	Sex	Municipality	$PRNT_{90}$ titer
A292	2016	53	F	Coari	1/80
A293	2016	41	M	Coari	Below LOD
A294	2016	36	F	Coari	Below LOD
A295	2016	22	F	Coari	Below LOD
A296	2016	30	F	Coari	Below LOD
A297	2016	57	F	Coari	Below LOD
A298	2016	44	M	Coari	Below LOD
A299	2016	22	F	Coari	Below LOD
A300	2016	60	F	Coari	Below LOD
A301	2016	45	M	Coari	Below LOD
A302	2016	23	F	Coari	Below LOD
A303	2016	18	M	Coari	Below LOD
A304	2016	67	F	Coari	1/80
A305	2016	22	F	Coari	Below LOD
A306	2016	75	M	Coari	Below LOD
A307	2016	45	M	Coari	Below LOD
A308	2016	43	F	Coari	Below LOD
A309	2016	57	M	Coari	Below LOD
A310	2016	56	F	Coari	Below LOD
A311	2016	28	F	Coari	Below LOD
A312	2016	NA	F	Coari	Below LOD
A313	2016	36	F	Coari	Below LOD
A314	2016	51	F	Coari	Below LOD
A315	2016	19	F	Coari	Below LOD
A316	2016	23	F	Coari	Below LOD
A317	2016	40	F	Coari	Below LOD
A318	2016	22	F	Coari	Below LOD
A319	2016	36	M	Coari	Below LOD
A320	2016	83	M	Coari	Below LOD
A321	2016	55	M	Coari	Below LOD
A322	2016	77	M	Coari	Below LOD
A323	2016	31	M	Coari	Below LOD
A324	2016	43	M	Coari	1/640
A325	2016	47	F	Coari	Below LOD
A326	2016	55	F	Coari	Below LOD
A327	2016	57	F	Coari	Below LOD
A328	2016	46	F	Coari	Below LOD
A329	2016	29	F	Coari	Below LOD
A330	2016	19	F	Coari	Below LOD
A331	2016	36	F	Coari	Below LOD
A332	2016	24	F	Coari	Below LOD
A333	2016	56	M	Coari	Below LOD
A334	2016	41	F	Coari	Below LOD
A335	2016	53	F	Coari	Below LOD
A336	2016	74	F	Coari	1/320
A337	2016	47	M	Coari	Below LOD
A338	2016	34	M	Coari	Below LOD
A339	2016	51	F	Coari	1/20
A340	2016	39	M	Coari	1/80
A341	2016	21	M	Coari	Below LOD
A342	2016	65	F	Coari	Below LOD
A343	2016	53	F	Coari	Below LOD
A344	2016	66	M	Coari	Below LOD
A345	2016	46	F	Coari	Below LOD
A346	2016	52	M	Coari	Below LOD
A347	2016	29	F	Coari	Below LOD
A348	2016	44	F	Coari	Below LOD
A349	2016	52	M	Coari	Below LOD
A350	2016	19	F	Coari	Below LOD
A351	2016	44	F	Coari	1/320
A352	2016	25	F	Coari	Below LOD
A353	2016	45	F	Coari	1/80
A354	2016	65	F	Coari	1/20
A355	2016	58	F	Coari	Below LOD
A356	2016	48	F	Coari	Below LOD
A357	2016	51	F	Coari	Below LOD
A358	2016	50	F	Coari	Below LOD
A359	2016	20	F	Coari	Below LOD
A360	2016	37	F	Coari	Below LOD

ID	Date	Age (years)	Sex	Municipality	$PRNT_{90}$ titer
A361	2016	53	M	Coari	Below LOD
A363	2016	26	F	Coari	Below LOD
A364	2016	56	M	Coari	Below LOD
A365	2016	36	M	Coari	Below LOD
A366	2016	26	M	Coari	Below LOD
A367	2016	36	F	Coari	Below LOD
A368	2016	23	F	Coari	Below LOD
A369	2016	42	M	Coari	Below LOD
A370	2016	18	F	Coari	Below LOD
A371	2016	42	F	Coari	Below LOD
A372	2016	36	F	Coari	1/40
A373	2016	25	F	Coari	Below LOD
A374	2016	34	F	Coari	Below LOD
A375	2016	49	F	Coari	1/40
A376	2016	41	F	Coari	Below LOD
A377	2016	24	F	Coari	Below LOD
A378	2016	27	F	Coari	Below LOD
A379	2016	28	F	Coari	Below LOD
A380	2016	24	F	Coari	Below LOD
A381	2016	38	F	Coari	Below LOD
A382	2016	27	F	Coari	Below LOD
A383	2016	48	F	Coari	Below LOD
A384	2016	49	F	Coari	Below LOD
A385	2016	84	M	Coari	1/160
A386	2016	34	F	Coari	Below LOD
A387	2016	33	M	Coari	Below LOD
A388	2016	36	F	Coari	Below LOD
A389	2016	43	F	Coari	1/40
A390	2016	51	F	Coari	Below LOD
A391	2016	67	F	Coari	Below LOD
A392	2016	53	M	Coari	1/80
A393	2016	35	F	Coari	Below LOD
A394	2016	60	F	Coari	Below LOD
A395	2016	26	M	Coari	Below LOD
A396	2016	49	F	Coari	1/640
A397	2016	18	M	Coari	Below LOD
A398	2016	26	F	Coari	Below LOD
A399	2016	43	F	Coari	Below LOD
A400	2016	20	F	Coari	Below LOD

*ID, identification. F, female. M, male. LOD, limit of detection. $PRNT_{90}$, plaque reduction neutralization test value 90.

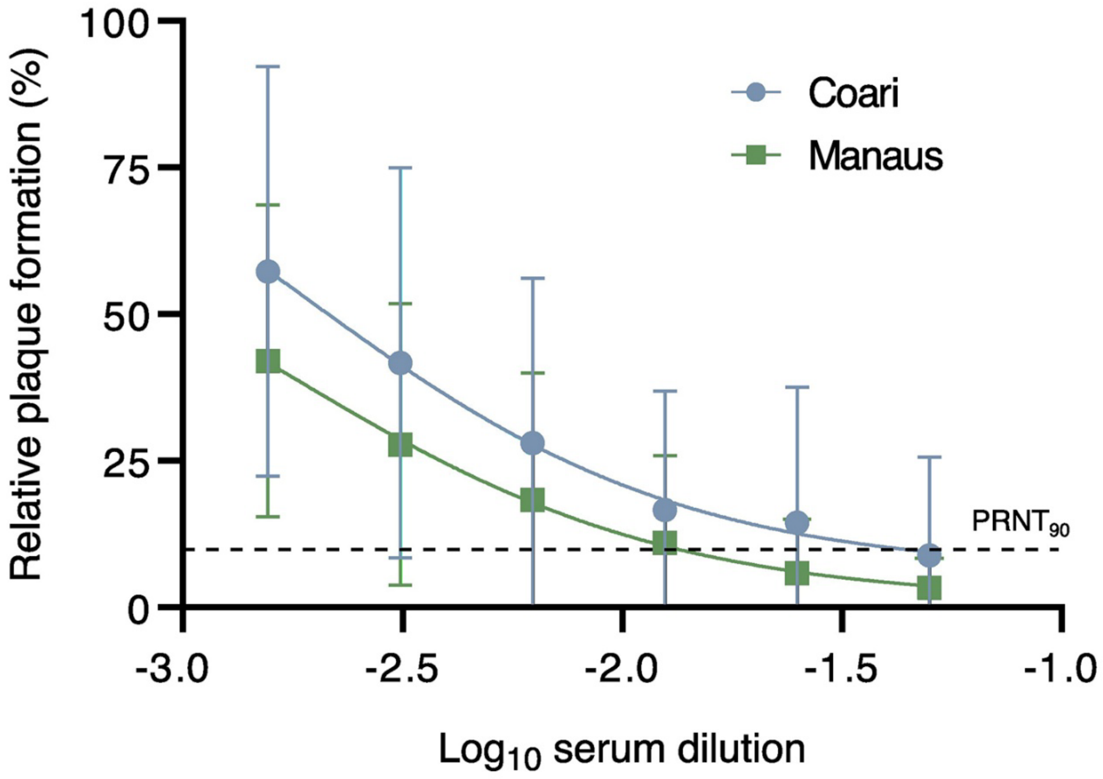

Appendix Figure. Neutralization of Oropouche virus strain BeAn19991 by $PRNT_{90}$. For relative plaque formation, each data point represents the mean of all serum samples for each group at each dilution level (shown as log_{10} serum dilution), and error bars represent standard deviation.
